# Management of the patient with failed hypospadias surgery presenting in adulthood

**DOI:** 10.12688/f1000research.11980.1

**Published:** 2017-10-26

**Authors:** Reem Aldamanhori, Christopher R. Chapple

**Affiliations:** 1 Department of Urology, Royal Hallamshire Hospital, Sheffield, UK; 2 Department of Urology, University of Dammam, Dammam, Saudi Arabia

**Keywords:** hypospadias, surgery, complications

## Abstract

The management of patients who have had complications of primary surgery for the resolution of a hypospadiac deformity remains a therapeutic challenge. Adults with complications following childhood hypospadias repairs are undoubtedly a difficult population to treat, as there is usually a cosmetic deformity, lower urinary tract symptoms, and resulting psychosexual consequences. A surgeon’s experience has been and still remains an important factor in determining subsequent surgical outcomes, particularly with more severe or complex cases. The purpose of this review is to evaluate the complications of hypospadias repair that present in adults and review published experience in treating them.

Hypospadias is seen in around one in 300 live births, making it the most prevalent congenital penile abnormality ^1^. Surgeons who perform hypospadias surgeries come from diverse specialty groups (pediatric surgery, plastic surgery, or urology). Consequently, preferred surgical technique and results vary significantly between different surgeons. Not surprisingly, the experience of the surgeon is also strongly associated with outcomes of hypospadias surgical repair ^2^, particularly when the length of the defect is greater. There is a steep learning curve for attaining surgical skill in primary hypospadias surgery, with limited long-term data on surgical outcomes, a dearth of information on patient-reported outcome measures, and limited reported expertise in redo surgery. Surgeons with subspecialist training in hypospadias surgery might be expected to achieve better outcomes with fewer complications than those without. If this is the case with primary hypospadias repair, then, inevitably, correcting the consequences of hypospadiac repair failure might be best managed in a specialized referral center.

In the absence of long-term follow-up and the limited number of centers providing transitional care from childhood to adulthood, there are limited long-term follow-up data. It can be speculated that the neourethra (a man-made tube) may fail to grow adequately in keeping up with the rest of the genital tissues during puberty, which may create a narrow or short tube. Due to rapid genital growth during puberty, some complications, including strictures, may not present until this time. In addition, it is assumed that the congenital lack of spongiosum covering the neourethra in those patients may not provide adequate vascular support to the urethra during this time. Similarly, it can be postulated that the trauma sustained during erection and sexual activity is not tolerated by the neourethra owing to lack of spongiosal support. The exact nature and range of complications in adulthood after childhood hypospadias repair remain poorly defined ^3^. In order to address this, pediatric urologists should follow-up hypospadias patients with severe hypospadias and those who underwent major repairs through puberty and again during the mid-teens until the completion of sexual maturity to detect latent urethral strictures.

Most studies on complications after hypospadias surgery have described only the complications occurring shortly after pediatric hypospadias surgery restricted to the childhood period. Roughly 30% of patients who undergo an initially successful repair in childhood end up with complications in adulthood, suggesting that the outcomes of repair may not be as durable as estimated by studies with short-term follow-up ^4^. Common complications that may require surgical resolution include meatal stenosis, urethrocutaneous fistulae, urethral stricture, persistent hypospadias, diverticula, and chordee. A further consideration, which is very important, is whether there is an associated skin condition such as balanitis xerotica obliterans (BXO), which would exclude the use of skin. The true incidence of urethral strictures is difficult to define because of a lack of series with long-term follow-up. Urethral strictures have been reported to be the second most common complication of hypospadias repair in the pediatric population with an incidence of 6.5% ^5^ and 10% of cases ^6^. Conversely, in a study of 74 adult patients who presented to a reconstructive urologist with complications related to prior hypospadias correction in childhood, urethral stricture disease was prevalent in 53%, and 57% of these patients underwent surgical correction ^3^. Although with any surgery some complications are unavoidable, there are certain factors that increase the chance of developing complications. These include poor surgical technique, postoperative infection, wound dehiscence, urine extravasation, hematoma, ischemia, and necrosis of the flap or graft used, leading to poor healing of the reconstructed tissue ^7^.

According to the American Academy of Pediatrics, the ideal age for hypospadias surgery is between 6 and 12 months. It is suggested that healing at this age occurs more promptly with less severe scarring ^8^. A study by Perlmutter *et al*. ^9^ reported considerably higher re-operation rate for hypospadias repair in infants older than 6 months than in younger ones, but this study did not take into account other potentially confounding variables, and this assertion has been challenged by a number of other authors ^10, 11^. A study by Snodgrass and colleagues suggested that increasing age does not indicate a greater risk for urethroplasty complications after hypospadias surgery ^10^. Furthermore, a recent study in the pediatric population by Snodgrass and Bush ^12^ reported that urethroplasty complications doubled in those undergoing a second hypospadiac urethroplasty compared with those undergoing a primary repair. This risk increased to 40% with three or more re-operations, with a 1.5-fold increase in complication rate with each surgery. ****

A patient with failed hypospadiac repair presenting in adulthood will have a more densely scarred penis, with less vascular and less pliable tissues to work with ^13^. Therefore, repeated attempts at surgical repair in those complicated cases are less likely to succeed. That is evident in a study by Hensle *et al*. ^14^, where in an adult group of patients who underwent hypospadias repair, complications were noted in 37.5% of patients who had no previous hypospadias surgery, 41.67% of patients who had undergone one or more procedures in childhood but in whom local tissue was relatively intact, and 63.6% of patients who underwent multiple unsuccessful hypospadias repairs with various degrees of penile deformity and loss of local tissue. Other studies concluded that although the number of surgeries for the correction of primary hypospadias may represent a risk factor for surgical failure, it was stricture length, not the number of previous operations needed for primary hypospadias repair, that was significantly associated with a risk of failure ^15^.

Patients with complications following hypospadias repair are a difficult population to treat. They could be left with deformities significantly worse than the primary congenital anomaly. The unanimous finding in the literature is that complications are significantly greater for re-operations than for primary repairs using the same surgical technique. This suggests the need for careful patient selection, modification of techniques for re-operation, and consideration of novel procedures not commonly used for primary repair ^16^.

Careful patient analysis is fundamental in choosing the technique, thereby attaining a successful salvage repair outcome ^17^. Careful and structured evaluation at the outpatient clinic is the key to the proper choice of surgical technique. Assessment of meatal site and size, stricture length, presence of surrounding tissue integrity and quality that might help in closure, size of the penis and glans, presence of fistulae, and degree of curvature are all important in deciding which repair technique should be used. Careful counseling of patients before surgery, taking into account their aspirations and giving them a realistic expectation of functional and sexual functional outcome, is essential. Even in seemingly simple cases, other issues are frequently seen, such as fistulae, scarring, recurrent chordee, and an abnormal meatus, often related to a small glans, and nearly always with a deficiency of the dartos layer ^18^, often with underlying associated psychosexual problems. Options for re-operative urethroplasty after failed hypospadias surgery include the use of either a flap or a graft to create a new urethra; this might be done in a single stage or usually over multiple stages depending on the extent of the repair needed. In particular, in addition to any underlying tissue deficiency, the presence of chordee, which has to be released, will often lead to proximal migration of the urethral meatus. Thus, treatment of strictures of the urethra and/or a meatal stenosis as a result of previous hypospadias surgery usually needs to be individualized.

There are several reported surgical techniques for the repair of complications after failed hypospadias repair in adults. The main principles of repair include excision of fibrosis to release any chordee and excision of scar tissue, which may include part, or even the whole length, of the previous urethroplasty, with any surrounding fibrous tissue. An adequate glans cleft is then created with the inlay of a graft or flap. The graft is secured to the corpora cavernosa from the proximal meatus out to the tip of the glans. If it is feasible, distal, and uncomplicated with good surrounding tissues and the urethroplasty segment is short, the creation of a neourethra and reconstruction of the glans can be done in one stage. If it is proximal and lengthy and needs a more extensive reconstruction and there is a deficiency of skin and subcutaneous tissues and dartos, it is best performed as a staged procedure with the inlay of a graft or flap and a subsequent closure procedure. Adequate time between staged procedures is needed for proper tissue healing and neovascularization to occur. This usually takes an average of 4–6 months. Patients are then evaluated in the outpatient clinic after the first stage to ensure that adequate healing has occurred with any significant fibrous contracture of the first stage and/or stenosis of the proximal urethral meatus. In some cases, a revision of the first stage might be necessary to deal with tissue contracture and/or inadequate take of the graft. Subsequently, to ensure optimal results, the final stage (closure) should include a watertight neourethral closure with good vascularized overlying tissue cover. In particular, care is taken not to overlap the suture lines in order to mitigate against fistula formation. The neourethra is reconstructed over a catheter or stent to divert the urine for 10–14 days with appropriate prophylactic antibacterial cover. In some cases, in our experience after the first stage procedure, a patient may decide to remain with the first stage rather than risk complications such as a meatal stenosis or chordee.

In a series of nine patients with adult urethral stricture disease after childhood hypospadias repair, three patients chose to receive repeated endoscopic treatments (visual internal urethrotomy and/or dilatations), and all of those cases had recurrence of the stricture ^19^. This confirms experience with urethrotomy for stricture disease in the penile urethra where urethrotomy cannot be recommended as a curative treatment. Reconstructive techniques including one-stage procedures (flaps or grafts) or multistage procedures with penile skin or buccal mucosa and even bladder mucosa have been described in the literature. There is controversy in the existing literature over which method should be chosen to treat this difficult situation, but in our view it is evident that therapy should be individualized to the patient following a careful evaluation, as noted above. In a series of primary repair, a comparison of flaps versus grafts has noted no significant difference in the complication rates when used to repair proximal hypospadias in children ^20^. Hypospadias repair with a bladder mucosa graft is feasible, although not popular, and long-term follow-up data are lacking. A series reported by Li *et al*. ^21^ showed their experience in using a free bladder mucosa graft in failed hypospadias in adolescents and adults with an 87.6% success and 12.4% complication rate after a follow-up ranging from 3 months to 2 years. In contrast, in another series in which bladder mucosa was used as a tube graft neourethra, graft contracture and fistula formed. Bladder mucosa for urethroplasty after hypospadias repair did not achieve the same results in both studies ^14^. The use of buccal mucosa as a substitute material in one-stage procedures showed complications requiring further surgery in between 24% ^22^ and 32% ^23^ of patients. Currently, the use of bladder mucosa has become far less common with increased use of oral mucosa. In another series, multistage procedures using buccal mucosa grafts reported complication rates of 10 to 35% ^24^. In a series reported by Tang *et al*. ^19^, four out of nine cases underwent urethroplasty with buccal mucosal grafts—single-stage repair was performed in two and two-stage repair was performed in another two—and two out of nine cases underwent salvage perineal urethrostomy. In a series by Barbagli *et al*. ^7^, 60 adults with previously failed hypospadias repair included 34 cases that underwent treatment for urethral stricture recurrence. There was an 87% success rate with one-stage repair without grafts, an 80% success rate with one-stage techniques using penile skin, and an 82% success rate with one-stage techniques using buccal mucosa. Multistage repairs delivered 50% success with penile skin and those with buccal mucosa provided 82% success. The one-stage direct repair without graft provided a higher success rate compared to the use of skin or buccal mucosa grafts, but these procedures were applied to simpler cases not requiring the use of substitution material for the urethral reconstruction. The one-stage techniques provided higher success rates when compared to multistage techniques, probably not surprisingly as the cases were less complex and required a shorter length of substitution. Those who needed multistage repairs showed a higher risk of failure because they had more severe strictures and extremely poor-quality native tissue than those considered for a single-stage repair. In multistage procedures, the buccal mucosa was superior to penile skin, showing a higher success rate.

Most studies state the outcome of the procedures without a clear description of inclusion/exclusion criteria used in decision making. In a reported series of hypospadias reoperation in children, an operation decision-making algorithm was centered on the presence or absence of an elastic urethral plate. When the urethral plate had no visible scarring, a TIP (tubularized incised plate) procedure was performed. If the urethral plate was previously excised but a skin strip without visible scarring remained in its location, one-stage inlay grafting was used. When the urethral plate, residual skin, or neourethra was visibly scarred, or there was a persistent ventral curvature greater than 30 degrees, hair in the neourethra, or suspicion of BXO, all grossly abnormal tissues were excised back to healthy urethra and a two-stage buccal graft urethroplasty was performed ^16^.

A significant portion of adults with hypospadias complications ultimately requires surgical intervention. Strictures in adults who had a hypospadias repair in childhood are usually complex; the neourethra usually contains skin from the earlier repair. Our experience over the last 35 years supports the observation that the longer the length of the previous repair, the more likely a stricture is to develop and the more complicated the stricture is likely to be ^25^. Urethral strictures in hypospadias failure can occur decades after initial hypospadias surgery and in view of their complexity often require multiple surgical procedures for their resolution ^19^. Although the impact of hypospadias repair almost unquestionably continues through adulthood, the majority of hypospadias studies lack long-term follow-up into adulthood ^26^. This is clearly shown by a survey of patients evaluated for failed hypospadias repair in a single reference center ^27^. Ideally, therefore, patients undergoing hypospadias surgery may receive lifelong follow-up but should at the very least be counseled to seek advice if they develop symptoms such as voiding difficulty and erectile deformity.

In adults with complications after failed hypospadias repair, no single procedure is considered standard of care. It is rare to see a patient who just needs a simple straightforward repair. Usually, taking down the previous urethroplasty and creating a new urethra, requiring more than one stage, is necessary. Owing to the variability of the deformities seen and the consequent functional abnormality, each patient should be evaluated as a separate entity and the treatment should be individualized. It is a challenging surgery, which needs comprehensive experience in the field of reconstruction. It must be borne in mind that any complex reconstructive procedure of this type has to be considered to comprise two phases—one has to take apart before putting back together—therefore, the surgeon’s experience is an important factor associated with a successful outcome ^4^. Nevertheless, all methods of urethral repair in this group of patients have the potential to fail; therefore, it is important to extend the follow-up after any such surgery, particularly in the more complex cases.

In the future, more attention needs to be placed on patient-reported outcome measures, taking into account cosmetic appearance, sexual function, and the patient’s wishes ^28, 29^.
